# Role and Function of A_2A_ and A_3_ Adenosine Receptors in Patients with Ankylosing Spondylitis, Psoriatic Arthritis and Rheumatoid Arthritis

**DOI:** 10.3390/ijms18040697

**Published:** 2017-03-24

**Authors:** Annalisa Ravani, Fabrizio Vincenzi, Alessandra Bortoluzzi, Melissa Padovan, Silvia Pasquini, Stefania Gessi, Stefania Merighi, Pier Andrea Borea, Marcello Govoni, Katia Varani

**Affiliations:** 1 Department of Medical Sciences, Pharmacology Unit, University of Ferrara, 44121 Ferrara, Italy; rvnnls@unife.it (A.R.); vncfrz@unife.it (F.V.); psqslv@unife.it (S.P.); gss@unife.it (S.G.); mhs@unife.it (S.M.); bpa@unife.it (P.A.B.); 2 Department of Medical Sciences, Section of Rheumatology, University of Ferrara and Azienda Ospedaliero Universitaria Sant’Anna, 44124 Cona, Ferrara, Italy; brtlsn@unife.it (A.B.); pdvmss@unife.it (M.P.); gvl@unife.it (M.G.)

**Keywords:** ankylosing spondylitis, psoriatic arthritis, rheumatoid arthritis, adenosine receptors, inflammation

## Abstract

Rheumatoid arthritis (RA), ankylosing spondylitis (AS) and psoriatic arthritis (PsA) are chronic inflammatory rheumatic diseases that affect joints, causing debilitating pain and disability. Adenosine receptors (ARs) play a key role in the mechanism of inflammation, and the activation of A_2A_ and A_3_AR subtypes is often associated with a reduction of the inflammatory status. The aim of this study was to investigate the involvement of ARs in patients suffering from early-RA (ERA), RA, AS and PsA. Messenger RNA (mRNA) analysis and saturation binding experiments indicated an upregulation of A_2A_ and A_3_ARs in lymphocytes obtained from patients when compared with healthy subjects. A_2A_ and A_3_AR agonists inhibited nuclear factor κ-light-chain-enhancer of activated B cells (NF-κB) activation and reduced inflammatory cytokines release, such as tumor necrosis factor-α (TNF-α), interleukin (IL)-1β and IL-6. Moreover, A_2A_ and A_3_AR activation mediated a reduction of metalloproteinases (MMP)-1 and MMP-3. The effect of the agonists was abrogated by selective antagonists demonstrating the direct involvement of these receptor subtypes. Taken together, these data confirmed the involvement of ARs in chronic autoimmune rheumatic diseases highlighting the possibility to exploit A_2A_ and A_3_ARs as therapeutic targets, with the aim to limit the inflammatory responses usually associated with RA, AS and PsA.

## 1. Introduction

The purine nucleoside adenosine has been identified as a major local tissue function regulator, particularly when cellular energy supply fails to meet demand [1]. Adenosine interacts with four different receptor subtypes, named as A_1_, A_2A_, A_2B_ and A_3_ adenosine receptors (ARs), that belong to the G-protein coupled receptor superfamily [2]. A_1_ and A_3_ receptors are negatively coupled to adenylate cyclase and exert an inhibitory effect on cyclic adenosine monophosphate (cAMP) production, whilst A_2A_ and A_2B_ receptors stimulate adenylate cyclase activity by increasing cAMP accumulation [3,4]. In the literature, several papers have suggested the involvement of adenosine in inflammatory status by A_2A_ and A_3_AR activation. In particular, it has been reported that many of the anti-inflammatory effects caused by the stimulation of A_2A_ and A_3_ARs are mediated by the suppression of pro-inflammatory cytokine release [5,6,7]. Furthermore, altered expression of A_2A_ and A_3_ARs has been found in different inflammation-related pathologies highlighting the potential role of these receptors as therapeutic targets in inflammatory diseases [8,9,10].

Rheumatoid arthritis (RA) represents one of the most important progressive, chronic and disabling systemic inflammatory diseases characterized by different joint destructive processes [11]. In particular, joint inflammation (i.e., synovitis) can lead to deformity associated with synovial proliferation and secretion of elevated levels of pro-inflammatory mediators such as cytokines and growth factors [12]. Pain and swelling are common joint symptoms that can begin at any age and causes fatigue and prolonged stiffness after rest, modifying quality of life and causing disability [13]. It is well-known that cytokines, such as tumor necrosis factor-α (TNF-α), interleukin (IL)-1 family and IL-6, have several significant activities in the context of the RA pathogenesis [14]. In particular, IL-6 interacts in complex ways with the cells involved in bone remodeling, indirectly promoting osteoclastogenesis and contributing to the severity of the radiological joint damage [15]. In previous studies our research group has found a significant upregulation of A_2A_ and A_3_ARs in blood cells from untreated RA patients and in methotrexate-treated RA patients, compared with healthy subjects [14]. In patients’ lymphocytes, it was shown that A_2A_ and A_3_AR activation inhibited the nuclear factor κ-light-chain-enhancer of activated B cells (NF-κB) pathway and diminished inflammatory cytokines such as TNF-α, IL-1β and IL-6. Moreover, A_2A_ and A_3_AR agonists mediated a reduction of different matrix metalloproteinases (MMP-1 and MMP-3) release [15]. Furthermore, we have demonstrated the direct involvement of A_2A_AR in RA pathogenesis based on their expression in relation to the time of pharmacological treatments with disease-modifying antirheumatic drugs (DMARDS) or different biological drugs. By using an adjuvant-induced rat model of arthritis, we previously reported that the treatment with an A_2A_AR agonist reduced the severity of clinical signs, decreased arthritis-associated pain, and increased the serum levels of the anti-inflammatory cytokine IL-10 [16]. The ability of the A_2A_AR agonist was also demonstrated in mice subjected to collagen-induced arthritis confirming its anti-inflammatory effect during chronic inflammation [17]. 

It is well-accepted that arthritis diseases also include spondyloarthritis, a family of chronic inflammatory diseases that share various common features such as axial arthritis and enthesitis. Ankylosing spondylitis (AS) and psoriatic arthritis (PsA) are among the most common diseases belonging to spondyloarthritis family. Ankylosing spondylitis is a chronic inflammatory disease that affects 1% of the general population, is regarded as one of the most severe types of spondyloarthropathy, it affects the spine and sacroiliac joints, causing debilitating pain and loss of mobility [18,19]. Psoriatic arthritis is a serious condition that causes joint destruction, disability, impaired quality of life and even increased mortality [20,21]. The first-line treatment is represented by nonsteroidal anti-inflammatory drugs (NSAIDs) in AS and by methotrexate in PsA. Increasing evidence suggests that many of the methotrexate effects are mediated by ARs activation. Indeed, it has been shown that methotrexate induces adenosine release in vitro, in animal models of inflammation and in patients with rheumatoid arthritis [22,23]. Furthermore, it is known that methotrexate selectively modulates the nuclear orphan receptor (NURR1) levels induced by inflammatory stimuli and growth factors in resident cell populations of synovial tissue [24]. The inhibitory effect of low-dose methotrexate on NURR1 activation is mediated through the A_2A_ARs, confirming the link between the immunomodulatory actions of methotrexate and adenosinergic system [25]. To date, a novel treatment to reduce clinical symptoms in PsA patients is represented by Apremilast, an orally available targeted inhibitor of phosphodiesterase-4 (PDE-4), the major enzyme class responsible for the hydrolysis of cAMP that modulates a wide array of inflammatory mediators implicated in PsA [26]. The role and the function of ARs during the course of chronic autoimmune rheumatic diseases are currently under study from different research groups because in the literature scarce information is available. 

In this study the involvement of ARs in chronic autoimmune rheumatic diseases represented by RA, AS and PsA was investigated. Of the four AR subtypes, A_2A_ and A_3_ARs were overexpressed in lymphocytes obtained from patients in comparison with those of healthy subjects. A_2A_ and A_3_AR agonists inhibited the activation of NF-κB, the release of typical pro-inflammatory cytokines and the metalloproteinases levels which are involved in the inflammatory responses in chronic autoimmune rheumatic diseases.

## 2. Results

### 2.1. Patients and Healthy Subjects

All patients enrolled in this study were recruited from the Rheumatology Section, Department of Medical Sciences, University of Ferrara, Italy. A total of 82 patients were included and divided in early-RA (ERA) untreated patients (*n* = 26), RA patients (*n* = 30), AS patients (*n* = 18) and PsA patients (*n* = 8). RA patients with established disease for more than 12 months fulfilled the American College of Rheumatology (ACR) 1987 criteria for rheumatoid arthritis. In particular, clinical data included: (i) age, disease duration, gender, serological parameters including rheumatoid factor (RF) and anti-citrullinated cyclic peptide (ACPA); (ii) Disease Activity Score evaluated in 28 joints (DAS28); (iii) functional status as assessed by Health Assessment Questionnaire (HAQ); (iv) pharmacological therapy as comedication with DMARDs such as methotrexate and leflunomide. The demographic, clinical and pharmacological details are listed in Table 1A. Biological naive, adult patients with AS classified according to the Assessment of SpondyloArthritis international Society (ASAS) criteria [27] and patients with PsA fulfilling the Classification of Psoriatic Arthritis (CASPAR) criteria [28] were included. Clinical data included: (i) age, disease duration, gender, human leukocyte antigen (HLA) B27 status, clinical presentation over disease history considering both the axial involvement and the presence of peripheral arthritis, dactylitis or entheseal pain; (ii) disease activity as assessed by the Bath Ankylosing Spondylitis Disease Activity Index (BASDAI) and DAS28; (iii) functional status as assessed by the Bath Ankylosing Spondylitis Functional Index (BASFI) and HAQ; (iv) pharmacological therapy as specific tumor necrosis factor-α inhibitor (TNFi), comedication with DMARDs. The demographic, clinical and pharmacological complete details are listed in Table 1B. This study has obvious limitations mainly related to the low number of patients analyzed, especially in the spondyloarthritis (AS and PsA). This could have induced an artefactual and chance inclusion of a low number of patients with positive serology both for RF and ACPA in the RA group and HLA B27+ in AS group. Healthy subjects (*n* = 80), matched for similar age to the cohort of the examined patients, were volunteers from Ferrara University Hospital Blood Bank. The study was approved by the local Ethics Committee (Approval No. 378, February 2010) of the University Hospital of Ferrara (Italy) and informed consent was obtained from each participant in accordance with the principles outlined in the Declaration of Helsinki.

### 2.2. A_2A_ and A_3_ARs Are Upregulated in Lymphocytes from Patients with Chronic Inflammatory Rheumatic Diseases

**Figure 1:**
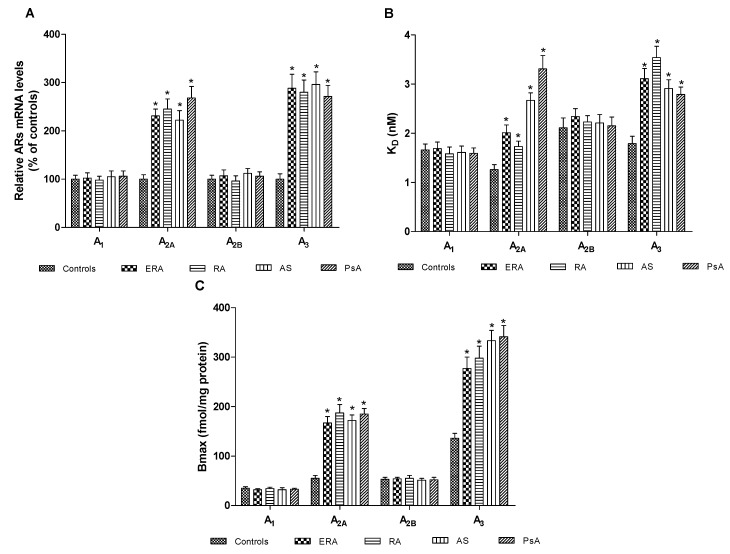
Adenosine receptors (ARs) A_2A_ and A_3_ARs are upregulated in patients’ lymphocytes with chronic inflammatory rheumatic diseases. (**A**) Relative ARs messenger RNA (mRNA) levels determined by real-time polymerase chain reaction (RT-PCR) in human lymphocytes from ERA (*n* = 26), RA (*n* = 30), AS (*n* = 18), PsA patients (*n* = 8) and control subjects (*n* = 80); (**B**) Affinity of A_1_, A_2A_, A_2B_, and A_3_ARs expressed as K_D_ values, in lymphocytes derived from ERA (*n* = 26), RA (*n* = 30), AS (*n* = 18) and PsA patients (*n* = 8) in comparison to control subjects (*n* = 80); (**C**) Density of A_1_, A_2A_, A_2B_, and A_3_ARs, expressed as the maximum specific binding (Bmax), in lymphocytes derived from ERA (*n* = 26), RA (*n* = 30), AS (*n* = 18) and PsA patients (*n* = 8) in comparison to control subjects (*n* = 80). Data are expressed as the mean ± SEM. * *p* < 0.01 vs. control group.

**Figure 2:**
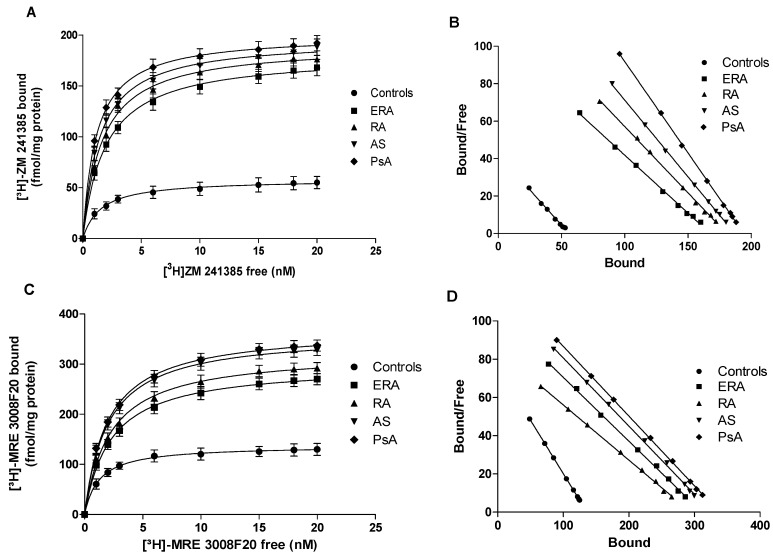
Saturation binding experiments in lymphocyte membranes from patients with chronic inflammatory rheumatic diseases. Saturation curves (**A**,**C**) and Scatchard plots (**B**,**D**) showing the binding of [^3^H]-ZM 241385 to A_2A_ARs (**A**,**B**) and the binding of [^3^H]-MRE 3008F20 to A_3_ARs (**C**,**D**) in lymphocyte membranes derived from 80 controls, 26 ERA patients, 30 RA patients, 18 AS patients and 8 PsA patients. Data are expressed as the mean ± SEM.

Figure 1A shows the A_1_, A_2A_, A_2B_ and A_3_AR mRNA levels determined by real-time polymerase chain reaction (RT-PCR) in human lymphocytes from healthy subjects and patients. Interestingly, only A_2A_ and A_3_AR messenger RNA (mRNA) expression in ERA, RA, AS, PsA patients was significantly increased. Figure 1B indicates the affinity (K_D_, nM) of A_1_, A_2A_, A_2B_ and A_3_ARs in lymphocyte membranes from ERA, RA, AS, PsA patients, compared to healthy subjects and shows that the affinity of A_2A_ and A_3_ARs was lower in patients than in controls (*p* < 0.01). The receptor density (Bmax, fmol/mg protein) of ARs was examined in lymphocyte membranes from patients and healthy subjects showing that the density of A_1_ and A_2B_ARs was not significantly different in patients compared to the control group (Figure 1C). Instead, A_2A_ and A_3_AR density was significantly increased in patients compared with healthy subjects (Figure 2). The K_D_ and Bmax values of ARs in lymphocyte membranes from control group and ERA, RA, AS, PsA patients are reported in Table 2.

### 2.3. Increased Potency of A_2A_ and A_3_ARs Agonists in Lymphocytes from Patients with Chronic Inflammatory Rheumatic Diseases

**Figure 3:**
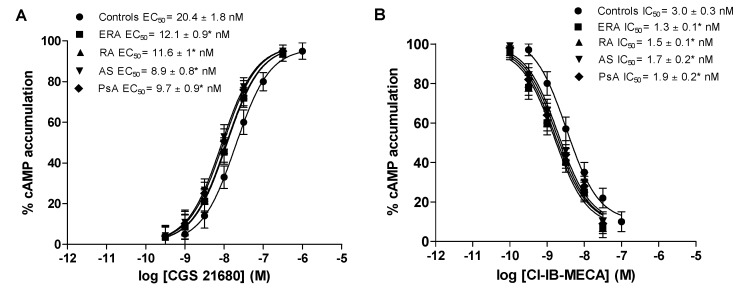
Increased potency of A_2A_ and A_3_AR agonists in patients’ lymphocytes with ERA, RA, AS and PsA diseases compared to control subjects. Concentration-response curves of CGS 21680 (**A**) or Cl-IB-MECA (**B**) on cyclic adenosine monophosphate (cAMP) assays in lymphocytes obtained from control subjects (*n* = 80), ERA (*n* = 26), RA (*n* = 30), AS (*n* = 18) and PsA patients (*n* = 8). Data are expressed as the mean ± SEM. * *p* < 0.01 vs. control group.

Figure 3 shows the concentration-response curves of typical A_2A_ and A_3_AR agonists such as CGS 21680 and Cl-IB-MECA, in lymphocytes from healthy subjects, ERA, RA, AS and PsA patients. The half maximal effective concentration (EC_50_) values of CGS 21680, indicating the concentration of the agonist eliciting 50% of maximal cAMP formation, were significantly lower in patients with chronic inflammatory rheumatic diseases than in control subjects, indicating an increased potency of CGS 21680 (Figure 3A). Similarly, Cl-IB-MECA showed higher potency in ERA, RA, AS and PsA patients, suggesting that the increase in A_3_ARs density was correlated with an increase of agonist potency (Figure 3B). 

### 2.4. A_2A_ and A_3_AR Agonists Reduces NF-κB Activation in Lymphocytes from the Examined Subjects

**Figure 4:**
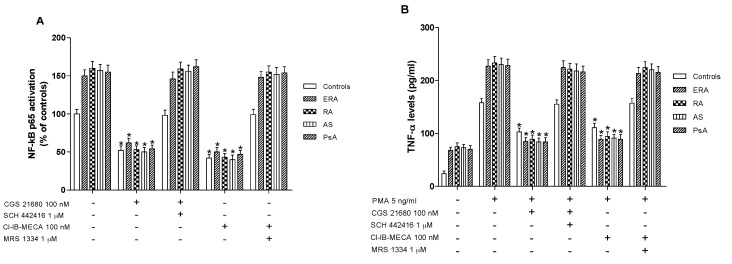
A_2A_ and A_3_AR stimulation reduced nuclear factor κ-light-chain-enhancer of activated B cells (NF-κB) activation and tumor necrosis factor-α (TNF-α) release. Effect of the A_2A_AR agonist CGS 21680 (100 nM) and A_3_AR agonist Cl-IB-MECA (100 nM) on NF-κB p65 subunit activation (**A**) or TNF-α release (**B**) in cultured lymphocytes from ERA (*n* = 26), RA (*n* = 30), AS (*n* = 18) and PsA patients (*n* = 8) in comparison to control subjects (*n* = 80). The A_2A_AR antagonist SCH 442416 (1 μM) and the A_3_AR antagonist MRS 1334 (1 μM) abrogated the effect of the agonists. Data are expressed as the mean ± SEM. * *p* < 0.01 vs. untreated cells (**A**); * *p* < 0.01 vs. phorbol myristate acetate (PMA)-stimulated cells (**B**).

Higher levels of activated NF-κB p65 were found in nuclear extract from cultured lymphocytes of ERA, RA, AS and PsA patients in comparison with control subjects (Figure 4A). CGS 21680, a well-known A_2A_AR agonist, was able to significantly inhibit NF-κB levels in cultured lymphocytes derived from the subjects investigated. This effect was abolished by using the selective A_2A_AR antagonist SCH 442416, suggesting the A_2A_-mediated effect of the agonist. Similar results were obtained with the A_3_AR agonist Cl-IB-MECA where its inhibitory effect on NF-κB activation was abrogated by A_3_AR antagonist MRS 1334. Interestingly, the inhibitory effect mediated by A_2A_ and A_3_AR agonists in patients with inflammatory rheumatic diseases was more marked than in control subjects (Figure 4A). 

### 2.5. A_2A_ and A_3_AR Activation Inhibits Cytokines Release from Lymphocytes of the Examined Subjects

**Figure 5:**
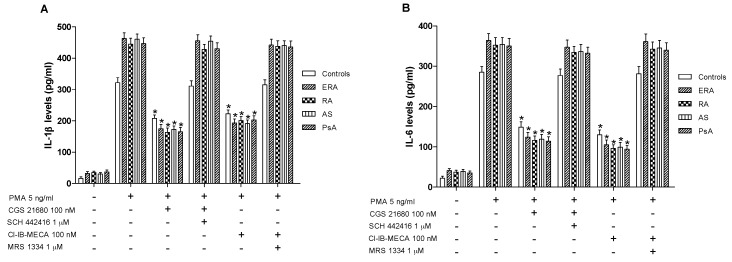
A_2A_ and A_3_AR activation reduced interleukin (IL)-1β and IL-6 release. Effect of the A_2A_AR agonist CGS 21680 (100 nM) and A_3_AR agonist Cl-IB-MECA (100 nM) on IL-1β (**A**) and IL-6 (**B**) release in cultured lymphocytes from ERA (*n* = 26), RA (*n* = 30), AS (*n* = 18) and PsA patients (*n* = 8) in comparison to control subjects (*n* = 80). The A_2A_AR antagonist SCH 442416 (1 μM) and the A_3_AR antagonist MRS 1334 (1 μM) blocked the effect of the agonists. Data are expressed as the mean ± SEM. * *p* < 0.01 vs. PMA-stimulated cells.

In cultured lymphocytes from patients and healthy subjects, the effect of A_2A_ and A_3_AR agonists and/or antagonists on TNF-α release was investigated. A marked release of TNF-α was observed in ERA, RA, AS and PsA patients compared to healthy subjects (Figure 4B). In addition, the stimulation of A_2A_AR with CGS 21680 mediated a significant inhibition of phorbol myristate acetate (PMA)-induced TNF-α release. The inhibitory effect of CGS 21680 was more evident in patients than in control subjects. Similar results were obtained through A_3_AR stimulation by using Cl-IB-MECA at the 100 nM concentration. The direct involvement of A_2A_ and A_3_ARs was demonstrated by using selective antagonists such as SCH 442416 and MRS 1334, respectively, which were able to completely abrogate the inhibitory effect mediated by the agonists. The effect of CGS 21680 or Cl-IB-MECA on IL-1β (Figure 5A) and IL-6 (Figure 5B) release was also studied in lymphocytes. Phorbol myristate acetate (5 ng/mL) induced a marked release of these pro-inflammatory cytokines and the treatment with A_2A_ and A_3_AR agonists resulted in a significant reduction of IL-1β and IL-6. In particular, the A_2A_AR agonist mediated a significant inhibition of IL-1β and IL-6 in ERA (62% and 66%, respectively), in RA (64% and 67%, respectively), in AS (62% and 67%, respectively) or in PsA patients (63% and 68%, respectively) (Figure 5A,B). Similar results were obtained by using A_3_AR stimulation on IL-1β and on IL-6 release. The inhibitory effect of A_2A_ or A_3_AR agonists was counteracted by the A_2A_ or A_3_AR antagonists SCH 442416 or MRS 1334 (1 μM), respectively.

### 2.6. A_2A_ and A_3_AR Agonists Reduced MMPs Activation in Monocytes from the Examined Subjects

**Figure 6:**
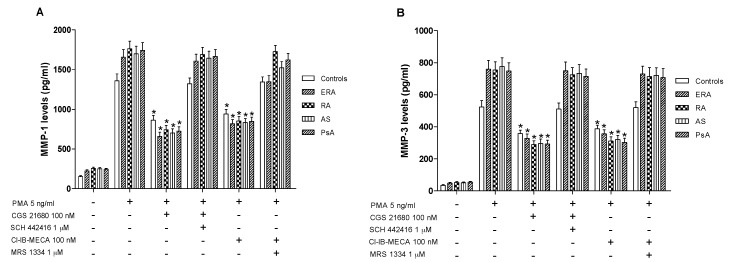
A_2A_ and A_3_AR activation reduced matrix metalloproteinases MMP-1 and MMP-3 production. Effect of the A_2A_AR agonist CGS 21680 (100 nM) and of the A_3_AR agonist Cl-IB-MECA (100 nM) on MMP-1 (**A**) or MMP-3 (**B**) production in cultured monocytes from ERA (*n* = 26), RA (*n* = 30), AS (*n* = 18) and PsA patients (*n* = 8) in comparison to control subjects (*n* = 80). The A_2A_AR antagonist SCH 442416 (1 μM) and the A_3_AR antagonist MRS 1334 (1 μM) blocked the effect of the agonists. Data are expressed as the mean ± SEM. * *p* < 0.01 vs. PMA-stimulated cells.

Monocytes were incubated with PMA (5 ng/mL) for 24 h inducing MMP-1 and MMP-3 protein production. Incubation of monocytes with CGS 21680 or Cl-IB-MECA (100 nM) caused an inhibition of MMP-1 production (Figure 6A). The inhibitory effect was more evident in inflammatory rheumatic disease patients respect to healthy subjects. Similar results were obtained evaluating the production of MMP-3, suggesting that these MMPs are closely associated with A_2A_ or A_3_AR modulation. The direct involvement of these AR subtypes was demonstrated with selective antagonists that blocked the inhibitory effect of the A_2A_ or A_3_AR agonists.

## 3. Discussion

It is widely accepted that adenosine-based systems are a key modulator of inflammatory responses affecting the release of several pro-inflammatory mediators implicated in the pathogenesis and progression of various pathologies such as rheumatic diseases [11]. To shed some light on the involvement of adenosine and its receptors in chronic inflammatory rheumatic diseases, this study was primarily aimed at investigating the expression of ARs in lymphocytes obtained from RA, AS and PsA patients in comparison with healthy subjects. mRNA analysis revealed a selective increase at a transcriptional level of A_2A_ and A_3_ARs in lymphocytes from arthritic patients. No significant differences were found between mRNA levels of A_1_ and A_2B_ARs in RA, AS and PsA patients compared to control subjects. The increase of A_2A_ and A_3_AR mRNA expression was accompanied by an upregulation of these receptor subtypes, as confirmed by saturation binding experiments. In particular, Bmax values increased by a range of 3.0- to 3.4-fold for A_2A_ARs and 2.0- to 2.5-fold for A_3_ARs in arthritis patients. These data confirmed our previous results obtained in RA patients where an upregulation of A_2A_ and A_3_ARs in lymphocytes was found [14,15,16]. 

The present study highlights, for the first time, the involvement of A_2A_ and A_3_ARs in two of the most common forms of spondyloarthritis, such as AS and PsA. Spondyloarthritis are a cluster of inflammatory conditions which share clinical genetic and pathophysiological characteristics [29]. It is worth noting that an overexpression of A_3_ARs was previously found in peripheral blood mononuclear cells derived from patients with RA, psoriasis and Crohn’s disease compared with healthy subjects [30]. All together these data consolidate the emerging role of ARs in autoimmune inflammatory diseases [11,22,31]. The effect on cAMP production of the A_2A_AR agonist CGS 21680 and of the A_3_AR agonist Cl-IB-MECA was tested in lymphocytes from RA, AS and PsA patients in comparison to healthy subjects. The increased potency of the two compounds found in patients affected by inflammatory rheumatic diseases under examination, together with the upregulation of A_2A_ and A_3_ARs, suggested the possibility of exploiting these receptor subtypes as therapeutic targets. To investigate the therapeutic potential of ARs modulation in arthritis diseases, the effect of specific agonists was studied in cultured lymphocytes from RA, AS and PsA patients on several inflammatory mediators such as NF-κB, cytokines and metalloproteinases. The transcription factor NF-κB is recognized as a key regulator of immune development, immune responses and inflammation [32]. It is well-established that the NF-κB pathway is essential both in acute inflammatory responses and in chronic inflammatory diseases, including arthritis-related diseases [33]. In recent work, a higher expression of NF-κB in peripheral blood leukocytes in patients with spondyloarthritis than in control group has been reported [34]. The results obtained in the present study revealed the capability of both the A_2A_AR agonist CGS 21680 and the A_3_AR agonist Cl-IB-MECA to reduce NF-κB p65 subunit activation in lymphocytes from RA, AS and PsA patients. Furthermore, the effect was more evident in patients than in healthy subjects, most likely due to the upregulation of A_2A_ and A_3_ARs. These data are consistent with those previously found in literature showing that A_2A_ and A_3_AR agonists are able to inhibit NF-κB activation both in vitro and in vivo. For instance, it has been shown that A_2A_AR activation inhibits osteoclast differentiation through the inhibition of NF-κB nuclear translocation, suggesting a mechanism by which adenosine could target bone destruction in inflammatory diseases [35]. Furthermore, it has been reported that the A_3_AR agonist CF502 inhibits the NF-κB signaling pathway in synoviocytes from RA patients and in adjuvant-induced arthritis rats [36]. 

The advancements in understanding the molecular and cellular mechanisms of chronic autoimmune rheumatic diseases have highlighted potential therapeutic of strategies aimed to inhibit the effects of upregulated cytokines and other pro-inflammatory mediators [6,37]. The beneficial effect of targeting pro-inflammatory cytokines is testified to by the clinical efficacy of monoclonal antibodies in biological drugs working against TNF-α, IL-6 or IL-1β in RA patients, whilst in AS and PsA, along with TNF-α, IL-12/23 and IL-17 axis is involved [38,39]. In this regard, two more human monoclonal antibodies have been approved for the treatment of PsA (ustekinumab, directed against IL-12/23) and secukinumab (directed against IL-17A) and the latter has also been approved for the AS treatment. In the present study, we have tested the potential inhibitory effect of A_2A_ and A_3_AR stimulation on the release of pro-inflammatory cytokines in lymphocytes obtained from patients with inflammatory rheumatic disorders in comparison to control subjects. The observed reduction of TNF-α, IL-6 and IL-1β achieved by using CGS 21680 or Cl-IB-MECA corroborated the anti-inflammatory effects of A_2A_ and A_3_AR activation and their potential therapeutic role, at least in RA. It is well-known that in RA inflammatory cytokines such as TNF-α, IL-6 and IL-1β, expressed locally in the articular joint, stimulate the production of MMPs. Increasing evidence has highlighted that MMP activity is upregulated in arthritic cartilage and synovial fluid. Though their pathogenetic role in spondyloarthritis is not fully defined, in both AS and PsA, MMPs have been advocated as potential biomarkers related to disease activity [40,41]. For these reasons, although there are different treatment options of varying efficacy for arthritis diseases, many alternatives are currently being explored, especially those that selectively inhibit some MMPs [42]. The results obtained in the present work indicated that both A_2A_ and A_3_AR stimulation inhibited MMP-1 and MMP-3 levels in monocytes for RA, AS and PsA patients and in control subjects. This effect was abrogated by selective antagonists for each receptor subtypes demonstrating that the inhibition of MMPs was mediated by A_2A_ and A_3_AR activation. In conclusion, our findings highlight the possibility of exploiting A_2A_ and A_3_ARs as therapeutic targets, with the aim of limiting the inflammatory processes usually associated with chronic autoimmune rheumatic diseases.

## 4. Materials and Methods

### 4.1. Sample Collection and Human Lymphocyte Preparation

Lymphocytes were isolated and prepared as previously described from the peripheral blood of control subjects, ERA, RA, AS and PsA patients. The isolation of blood cells started no later than 3–4 h after the samples had been taken. The blood was supplemented with 6% (by weight) Dextran T500 solution (Sigma, St. Louis, MO, USA) and erythrocytes were allowed to settle down for 60 min. Leukocytes were centrifuged for 15 min at 100× *g* and remaining erythrocytes were lysed in distilled water at 4 °C. Cells were pelletted by centrifugation for 5 min at 250× *g*, suspended in Krebs-Ringer phosphate buffer and layered onto 10 mL of Fycoll-Hypaque (GE Healthcare, Little Chalfont, UK). After centrifugation, mononuclear cells were washed in 0.02 M phosphate-buffered saline at pH 7.2 containing 5 mM MgCl_2_ and 0.15 mM CaCl_2_. Finally, they were decanted into culture flask and placed in a humidified incubator (5% CO_2_) for 2 h at 37 °C. This procedure, aimed at removing monocytes, which adhere to the culture flasks, resulted in a purified lymphocyte preparation containing at least 99% small lymphocytes identified by morphological criteria. To obtain membrane suspensions, cell fractions were centrifuged in hypotonic buffer at 20,000× *g* for 10 min. The resulting pellet was re-suspended in Tris HCl 50 mM buffer pH 7.4 containing 2 UI/mL adenosine deaminase (Sigma) and incubated for 30 min at 37 °C. The suspension was then centrifuged again at 40,000× *g* for 10 min and the final pellet was used for radioligand binding experiments. The protein concentration was determined by a Bio-Rad method with bovine albumine as reference standard [14].

### 4.2. Real-Time Quantitative Polymerase Chain Reaction Experiments

Total cytoplasmic RNA was extracted by the acid guanidinium thiocyanate phenol method. Quantitative RT-PCR assays of A_1_, A_2A_, A_2B_ and A_3_AR mRNAs were carried out using gene-specific fluorescently labelled TaqMan MGB probe (minor groove binder) in a ABI Prism 7700 Sequence Detection System (Applied Biosystems, Warrington Cheshire, UK). For the RT-PCR of A_1_, A_2A_, A_2B_ and A_3_ARs the Assays-on-Demand^TM^ Gene Expression Products NM 000674, NM 000675, NM 000676 and NM 000677 were used, respectively. For the RT-PCR of the reference gene, the endogenous control human β-actin kit was used, and the probe was fluorescent-labeled with VICTM (Applied Biosystems, Monza, Italy) [14].

### 4.3. Saturation Binding Experiments to A_1_, A_2A_, A_2B_ and A_3_ARs

Saturation binding experiments to A_1_ARs were carried out with the use of [^3^H]-DPCPX ([^3^H]-1,3-dipropyl-8-cyclopentyl-xanthine, specific activity 120 Ci/mmol, Perkin Elmer Life and Analytical Sciences, Boston, MA, USA) as radioligand [14]. Human lymphocyte membranes (60 μg of protein/assay) with 8 to 10 concentrations of [^3^H]-DPCPX (0.01–20 nM) were incubated for 90 min at 25 °C. Non-specific binding was determined in the presence of 1 μM DPCPX. Saturation binding to A_2A_ARs was performed with the use of [^3^H]-ZM 241385 ([^3^H]-4-(2-[7-amino-2-(2-furyl)[1,2,4]-triazolo[2,3-a][1,3,5] triazin-5-ylamino] ethyl) phenol, specific activity 27 Ci/mmol, Biotrend, Cologne, Germany), as radioligand [14]. Cell membranes (60 μg of protein/assay) were incubated for 60 min at 4 °C with various concentrations (0.01–20 nM) of [^3^H]-ZM 241385. Non-specific binding was determined in the presence of 1 μM ZM 241385. Saturation binding experiments to A_2B_ARs were performed by using [^3^H]-MRE 2029F20 ([^3^H]-*N*-benzo[1,3]dioxol-5-yl-2-[5-(2,6-dioxo-1,3-dipropyl-2,3,6,7-tetrahydro-1H-purin-8-yl)-1-methyl-1H-pyrazol-3-yl-oxy]-acetamide, specific activity 123 Ci/mmol, GE Healthcare, UK) as radioligand [14]. Cell membranes (80 μg of protein/assay) and [^3^H]-MRE 2029F20 (0.01–30 nM) were incubated for 60 min at 4 °C and non-specific binding was determined in the presence of 1 μM MRE 2029F20. Saturation binding experiments to A_3_ARs were carried out using [^3^H]-MRE 3008F20 ([^3^H]-5*N*-(4-methoxyphenylcarbamoyl) amio-8-propyl-2-(2-furyl) pyrazolo [4,3-e]-1,2,4-triazolo [1,5-c]pyrimidine, specific activity 67 Ci/mmol, GE Healthcare, UK) as radioligand [14]. The membranes (80 μg of protein/assay) with [^3^H]-MRE 3008F20 (0.01–30 nM) were incubated at 4 °C for 150 min and MRE 3008F20 1 μM was used to evaluate non-specific binding. Bound and free radioactivity were separated in a Brandel cell harvester (Brandel, Gaithersburg, MD, USA) by filtering the assay mixture through Whatman GF/B glass fiber filters (Whatman. Kent, UK). The filter-bound radioactivity was counted in a 2810 TR liquid scintillation counter (Perkin Elmer, Boston, MA, USA).

### 4.4. Lymphocyte Cell Culture

Isolated lymphocytes from control subjects, ERA, RA, AS and PsA patients were suspended at a density of 106 cells/mL in RPMI 1640 medium supplemented with 2% fetal bovine serum (Euroclone, Milan, Italy) and seeded into 24-well plates (1 mL/well). Cells were then preincubated for 15 min with 100 nM of CGS 21680 (4-[2-[[6-amino-9-(*N*-ethyl-β-d-ribo-furanuronamidosyl)-9H-purin-2-yl]amino] ethyl]benzene propanoic, Sigma) or Cl-IB-MECA (N6-(3-iodo-benzyl)-2-chloro-adenosine-5′-*N*-methyluronamide, Sigma) in the absence and in the presence of selected A_2_A or A_3_AR antagonists such as SCH 442416 (2-(2-furanyl)-7-[3-(4-methoxyphenyl)propyl]-7*H*-pyrazolo[4,3-e][1,2,4] triazolo [1,5-c]pyrimidin-5-amine, Tocris Bioscience, Bristol, UK) or MRS 1334 (1,4-dihydro-2-methyl-6-phenyl-4-(phenylethynyl)-3,5-pyridinedicarboxylic acid 3-ethyl-5-[(3-nitrophenyl)methyl] ester, Tocris Bioscience) at 1 μM concentration, respectively. Lymphocytes were then activated with 5 ng/mL PMA and incubated for 24 h [15]. At the end of incubation, the cell suspension was collected and centrifuged at 1000× *g* for 10 min at 4 °C. Then, the supernatants or cell pellets were used for enzyme-linked immunosorbent assay (ELISA) assays or nuclear extract preparation, respectively. 

### 4.5. Measurement of cAMP Levels

In lymphocytes, the potency of the well-known A_2A_ and A_3_ adenosine agonists CGS 21680 and Cl-IB-MECA (0.1 nM–1 μM) was investigated. Cells were seeded in 96-well white half-area microplate (Perkin Elmer, Boston, MA, USA) in a stimulation buffer composed of Hank Balanced Salt Solution, 5 mM HEPES, 0.5 mM Ro 20-1724, 0.1% BSA. Forskolin (1 μM) was used to stimulate adenylyl cyclase activity after the addition of Cl-IB-MECA. cAMP levels were then quantified by using the AlphaScreen cAMP Detection Kit (Perkin Elmer) following the manufacturer’s instructions. At the end of the experiments, plates were read with the Perkin Elmer EnSight Multimode Plate Reader. 

### 4.6. NF-κB Activation in Human Cultured Lymphocytes

Nuclear extracts from human cultured lymphocytes of the examined patients were prepared by using a nuclear extract kit (Active Motif, Carlsbad, CA, USA) following the manufacturer’s instructions. Activation of NF-κB was evaluated by detecting phosphorylated p65 proteins in nuclear extracts by using the TransAM NF-κB kit (Active Motif). Phosphorylated NF-κB subunits specifically bind to the immobilized oligonucleotides containing the NF-κB consensus site (5′-GGGACTTTCC-3′). The primary antibody used to detect NF-κB recognized an epitope in the subunits that is accessible only when it is activated and bound to its DNA target. A horseradish peroxidase (HRP)-conjugated secondary antibody provided a sensitive colorimetric readout that was quantified by spectrophotometry at 450 nm wavelength with the Perkin Elmer EnSight Multimode Plate Reader [15].

### 4.7. Pro-Inflammatory Cytokine Release in Cultured Lymphocytes

TNF-α levels were measured in human cultured lymphocytes after the treatment described above by using highly sensitive TNF-α ELISA (R & D Systems, Minneapolis, MN, USA) according to the manufacturer’s instructions. Pro-inflammatory cytokine (IL-1β and IL-6) levels were determined with a quantitative sandwich ELISA kit (R & D Systems, Minneapolis, MN, USA) according to the manufacturer’s instructions [15]. The reaction was developed with streptavidin-horseradish peroxidase and optical density was read at 450 nm wavelength with the Perkin Elmer EnSight Multimode Plate Reader.

### 4.8. Measurement of Total MMP-1 and MMP-3 Release in Cultured Monocytes

To obtain human monocytes, peripheral blood mononuclear cells were seeded in petri dishes at the density of 10^6^/mL. The cells were allowed to adhere to plastic tissues and non-adherent cells (lymphocytes) were removed. In cultured monocytes, MMP levels were measured after the treatment described above by using the corresponding quantitative sandwich ELISA kit (R & D Systems) according to the manufacturer’s instructions [15]. Briefly, the assay systems measure natural and recombinant human active and pro-MMPs (total MMPs).

### 4.9. Statistical Analysis

Dissociation equilibrium constants for saturation binding, affinity, or K_D_ values, as well as the maximum densities of specific binding sites (Bmax), were calculated for a system of one- or two-binding site populations by means of a non-linear curve fitting using GraphPad Prism software version 6.0 (GraphPad Software, Inc., San Diego, CA, USA). All data are reported as mean ± SEM of different independent experiments as indicated in the Results section or in the Figure legends. Analysis of data was performed by one-way analysis of variance (ANOVA). Differences between the groups were analyzed with Dunnett’s test and were considered significant at a value of *p* < 0.01.

## Figures and Tables

**Table 1:** Clinical and demographic features of the study population, including healthy subjects, patients with rheumatoid arthritis (including early forms) (**A**) and spondyloarthritis (**B**).

**No. Healthy Subjects**	**80**
No. female/male	59/21
Age, mean ± SEM years	54.1 ± 5.6
**A. Rheumatoid arthritis**	
**No. Early rheumatoid arthritis (ERA)**	**26**
No. female/male	21/5
Age, mean ± SEM years	52.4 ± 2.5
Disease duration (months)	10.3 ± 3.1
Rheumatoid Factor	6/26 (23%)
ACPA	10/26 (38%)
DAS28, mean ± SEM	3.2 ± 0.2
HAQ, mean ± SEM	1.2 ± 0.1
*Concomitant DMARDs or TNF inhibitors*	0
**No. Rheumatoid arthritis (RA)**	**30**
No. female/male	26/4
Age, mean ± SEM years	53.8 ± 2.9
Disease duration (months)	74.3 ± 8.4
Rheumatoid Factor	9/30 (30%)
ACPA	13/30 (43%)
DAS28, mean ± SEM	3.4 ± 0.2
HAQ, mean ± SEM	1.3 ± 0.1
*Concomitant DMARDs:*	
Methotrexate (10–15 mg/week)	30 100%)
**B. Spondyloarthritis**	
**No. Seronegative spondyloarthritis (including AS and PsA)**	**26**
No. female/male	7/19
Age, mean ± SEM years	38.4 ± 2.4
Disease duration (months)	121 ± 14
**Ankylosing spondylitis (AS)**	18 (69.3%)
Axial involvement (only)	14/18 (77.8%)
Entheseal involvement ^1^	8/18 (50%)
Axial and peripheral involvement	4/18 (22.2%)
HLA B27 positive	8/18 (44.5%)
**Psoriatic arthritis (PsA)**	8 (30.7%)
Peripheral involvement (only)	5/8 (62.5%)
Axial and peripheral involvement	3/8 (37.5%)
Entheseal involvement ^1^	4/8 (50%)
Dactylitis	1/8 (12.5%)
*Clinimetric measures:*	
DAS28 ^2^, mean ± SEM	3.9 ± 0.3
HAQ ^2^, mean ± SEM	0.8 ± 0.1
BASDAI ^3^, mean ± SEM	5.2 ± 0.5
BASFI ^3^, mean ± SEM	0.8 ± 0.3
*Concomitant DMARDs:*	
Methotrexate (10–15 mg/week)	1 (3.8%)
Leflunomide	1 (3.8%)
*TNF inhibitors:*	
Infliximab in AS/PsA	10/2 (55.5%/25%)
Adalimumab in AS/PsA	8/4 (44.5%/50%)
Etanercept in AS/PsA	0/2 (0%/25%)

ERA: Early-rheumatoid arthritis; RA: Rheumatoid arthritis; ACPA: Anti-citrullinated protein antibodies; ^1^ Entheseal involvement includes: Patellar and quadriceps tendons, Achilles tendon, plantar fascia, medial and lateral epicondyle; ^2^ For patients with peripheral joint involvement (*n* = 12); ^3^ For patients with axial involvement (*n* = 21); DAS28: Disease activity score evaluated in 28 joints; HAQ: Health assessment questionnaire; BASDAI: Bath Ankylosing Spondylitis Disease Activity Index; BASFI: Bath Ankylosing Spondylitis Functional Index; DMARDs: Disease-modifying antirheumatic drugs; TNF: tumor necrosis factor; HLA: Human leukocyte antigen; SEM: standard error of the mean.

**Table 2:** Adenosine receptor binding parameters in lymphocytes from patients with chronic autoimmune rheumatic diseases in comparison with healthy subjects.

	A_1_ARs - K_D_ (nM)	A_2A_ARs - K_D_ (nM)	A_2B_ARs - K_D_ (nM)	A_3_ARs - K_D_ (nM)
	Bmax (fmol/mg protein)	Bmax (fmol/mg protein)	Bmax (fmol/mg protein)	Bmax (fmol/mg protein)
Healthy subjects	1.66 ± 0.12	1.26 ± 0.10	2.11 ± 0.20	1.79 ± 0.15
(*n* = 80)	35 ± 3	55 ± 6	53 ± 4	136 ± 10
ERA patients	1.69 ± 0.13	2.01 ± 0.16 *	2.34 ± 0.16	3.11 ± 0.21 *
(*n* = 26)	32 ± 2	167 ± 13 *	54 ± 3	277 ± 23 *
RA patients	1.58 ± 0.14	1.73 ± 0.11 *	2.23 ± 0.13	3.54 ± 0.23 *
(*n* = 30)	34 ± 3	175 ± 17 *	55 ± 6	298 ± 24 *
AS patients	1.61 ± 0.13	2.67 ± 0.15 *	2.21 ± 0.17	2.91 ± 0.18 *
(*n* = 18)	32 ± 4	185 ± 11 *	51 ± 4	333 ± 21 *
PsA patients (*n* = 8)	1.59 ± 0.11	3.31 ± 0.27 *	2.15 ± 0.18	2.79 ± 0.15 *
	33 ± 2	192 ± 11 *	52 ± 5	341 ± 23 *

Data are expressed as the mean ± SEM. Differences were considered significant at a value of * *p* < 0.01 vs. healthy controls.

## Abbreviations

RA	Rheumatoid arthritis
AS	Ankylosing spondylitis
PsA	Psoriatic arthritis
ARs	Adenosine receptors
NF-κB	Nuclear factor κ-B
TNF-α	Tumor necrosis factor α
IL	Interleukin
MMP	Metalloproteinases
cAMP	Cyclic adenosine monophosphate
DMARDS	Disease-modifying antirheumatic drug
NSAIDs	Nonsteroidal anti-inflammatory drugs
NURR1	Nuclear orphan receptor
PDE-4	Phosphodiesterase-4
ACR	American College of Rheumatology
RF	Rheumatoid factor
ACPA	Anti-citrullinated cyclic peptide
DAS28	Disease Activity Score evaluated in 28 joints
HAQ	Health Assessment Questionnaire
ASAS	Assessment of SpondyloArthritis international Society
CASPAR	Classification of Psoriatic Arthritis
BASDAI	Bath Ankylosing Spondylitis Disease Activity Index
BASFI	Bath Ankylosing Spondylitis Functional Index
RT-PCR	Real-time polymerase chain reaction
PMA	Phorbol myristate acetate
